# Why European and United States drug regulators are not speaking with one voice on anti-influenza drugs: regulatory review methodologies and the importance of ‘deep’ product reviews

**DOI:** 10.1186/s12961-017-0259-8

**Published:** 2017-11-09

**Authors:** Shai Mulinari, Courtney Davis

**Affiliations:** 1 Department of Sociology, Faculty of Social Sciences, Lund University, Box 117, 221 00 Lund, Sweden; 2 Department of Global Health and Social Medicine, Faculty of Social Science and Public Policy, Kings College London, London, United Kingdom

**Keywords:** Neuraminidase inhibitor, Influenza, Meta-analysis, United States Food and Drug Administration, European Medicines Agency

## Abstract

**Background:**

Relenza represents the first neuraminidase inhibitor (NI), a class of drugs that also includes the drug Tamiflu. Although heralded as breakthrough treatments in influenza, NI efficacy has remained highly controversial. A key unsettled question is why the United States Food and Drug Administration (FDA) has approved more cautious efficacy statements in labelling than European regulators for both drugs.

**Methods:**

We conducted a qualitative analysis of United States and European Union regulatory appraisals for Relenza to investigate the reasons for divergent regulatory interpretations, pertaining to Relenza’s capacity to alleviate symptoms and reduce frequency of complications of influenza.

**Results:**

In Europe, Relenza was evaluated via the so-called national procedure with Sweden as the reference country. We show that FDA reviewers, unlike their European (i.e. Swedish) counterpart, (1) rejected the manufacturer’s insistence on pooling efficacy data, (2) remained wary of subgroup analyses, and (3) insisted on stringent statistical analyses. These differences meant that the FDA was less likely to depart from prevailing regulatory and scientific standards in interpreting trial results. We argue that the differences are explained largely by divergent institutionalised review methodologies, i.e. the European regulator’s reliance on manufacturer-compiled summaries compared to the FDA’s examination of original data and documentation from trials.

**Conclusions:**

The FDA’s more probing and meticulous evaluative methodology allowed its reviewers to develop ‘deep’ knowledge concerning the clinical and statistical facets of trials, and more informed opinions regarding suitable methods for analysing trial results. These findings challenge the current emphasis on evaluating regulatory performance mainly in terms of speed of review. We propose that persistent uncertainty and knowledge deficits regarding NIs could have been ameliorated had regulators engaged in the public debates over the drugs’ efficacy and explained their contrasting methodologies and judgments. Regulators use major resources to evaluate drugs, but if regulators’ assessments are not effectively disseminated and used, resources are used inefficiently.

## Background

The present study aims to investigate and explain conflicting assessments of the anti-influenza drug Relenza (zanamivir) made by United States and European regulators. Marketed since 1999 by the British drug firm Glaxo Wellcome (GW) (subsequently GlaxoSmithKline), Relenza represents the first so-called neuraminidase inhibitor (NI), a class of drugs that also includes the drug Tamiflu (oseltamivir). Although heralded as breakthrough treatments [1], the efficacy of Relenza and Tamiflu have remained highly controversial, especially after many countries invested huge sums of money stockpiling the drugs to safeguard public health in the event of a major influenza outbreak [2, 3]. In particular, controversies have surrounded the incongruent results from meta-analyses and systematic reviews authored or ordered by a variety of actors, including Health Technology Assessment bodies [4], investigators employed or financed by the drug manufacturers [5–7], and non-industry funded academics [8, 9]. Whilst meta-analyses and systematic reviews are intended to serve as key tools for evidence-based medicine [10], in the case of the NIs, they have been a source of confusion and contestation [11]. For example, meta-analytic estimates of the drugs’ capacity to reduce the amount of time people experience symptoms of influenza have ranged from less than 1 day [5] to up to 2 days [4]. As a consequence, policy recommendations have also varied, with some authors and organisations endorsing the use of NIs and advocating national stockpiling, while others – arriving at more conservative estimates of efficacy – discouraged it [11, 12].

Of particular note were the conflicting statements about the efficacy of NIs made by three of the most prominent public health bodies in the United States, namely the Centres for Disease Control and Prevention, the Department of Health and Human Services, and the United States Food and Drug Administration (FDA) [2], leading one commentator to ask “*Why aren’t the United States Centers for Disease Control and Food and Drug Administration speaking with one voice on flu?*” [13]. In the midst of these high-profile controversies, and central to this study’s rationale, researchers and commentators expressed bewilderment over the FDA having approved much more cautious (and sometimes directly contradictory) efficacy statements than European regulators, as evidenced by the description of treatment effects printed in regulator-approved drug labels [2, 8]. For example, United States labels state that both Relenza and Tamiflu have not been proven to reduce complications of influenza whilst European labels claim the opposite. Similarly, the FDA maintains that Relenza and Tamiflu have not been shown to attenuate illness in patients at high risk of complications, but European labels claim the opposite. These conflicting regulatory statements regarding treatment benefits have moved to the centre of debates because a major argument for stockpiling NIs was the anticipated effect on reducing complications of influenza linked to morbidity and death, and their assumed therapeutic benefits in some high-risk patients [2]. Yet, curiously, the reasons underlying United States and European regulators’ divergent interpretations of NI efficacy remain unexplored and unexplained [8].

Below, we investigate cross-national regulatory divergences as they pertain to Relenza. Methodologically, the choice of Relenza, instead of Tamiflu, for this analysis is based on previous research showing how ‘first-in-class’ drugs may create regulatory precedents [14]; indeed, United States and European regulators’ divergent conclusions are similar for Relenza and Tamiflu [8], suggesting a common cause. Our analysis traces links between regulatory agency resources, institutionalised review methodologies, disciplinary expertise, the methods and standards used by agency reviewers for evaluating trial results, and regulatory outcomes in the form of particular statements about efficacy. In brief, we argue that the FDA’s more probing and meticulous evaluative methodology allowed its reviewers to develop ‘deep’ knowledge concerning the clinical and statistical facets of trials, and more informed opinions regarding suitable methods for analysing trial results. We also propose that persistent uncertainty and knowledge deficits regarding NIs could have been ameliorated had regulators engaged in the public debates and explained their contrasting methodologies and judgments.

## Methods

We used a qualitative, cross-national comparative methodology [15] to investigate divergent interpretations of Relenza’s capacity to (1) alleviate symptoms and (2) reduce the frequency of complications of influenza. In the European Union, Relenza was evaluated under the mutual recognition procedure [16], with Sweden as the reference member state. We asked for and obtained relevant regulatory documents from the Medical Products Agency (MPA) under the Swedish Public Access to Information and Secrecy Act, including company submissions to the MPA and regulatory reviews and product labels (also known as the Summary of Product Characteristics in Europe). In the United States, Relenza was evaluated by the FDA’s Division of Antiviral Drug Products (DAVP). From the FDA webpage we downloaded publically available regulatory documents. This included the DAVP’s medical and statistical reviews and product labels, but also memos from meetings and discussions held with the manufacturer. Our research also involved extensive documentary data collection and analysis, including review of the scientific and ‘grey’ literature (e.g. government or public body reports) on NIs.

While the analysis of FDA and MPA regulatory documents form the backbone of this study, we also conducted a number of semi-structured interviews to ensure proper understanding of the clinical research and regulatory context relevant to NIs. This included interviewing a former FDA reviewer who had participated in evaluating the Relenza submission, as well as a senior MPA statistician who was a long-time member of the MPA Board that scrutinised all regulatory assessments, including at the time of Relenza evaluation. We also interviewed three academics that had meta-analysed the Relenza trial programme. Despite repeated attempts, representatives of GlaxoSmithKline and the MPA clinical assessor who evaluated Relenza declined to be interviewed or respond to specific queries. We acknowledge this may be a limitation of the study.

## Results

### The Relenza submission

This section provides an overview of the evidence GW submitted to United States and Swedish regulatory authorities most relevant to deliberations about Relenza’s capacity to alleviate symptoms and reduce frequency of complications of influenza. The clinical development programme for Relenza included three phase III (‘pivotal’) studies carried out during the 1997/1998 influenza season. One was conducted in Europe [17], one in the Southern Hemisphere (primarily Australia) [18], and one in North America (primarily the United States) (unpublished). Results from a number of phase II trials were also included in the application as supporting evidence. In addition to these treatment trials, the applications included one (to the FDA) and seven (to the MPA) influenza prevention (or prophylaxis) studies. The explanation for this difference was that GW sought approval for both indications in Sweden, but only the treatment indication in the United States (only the treatment indication is discussed in this text).

To be recruited into Relenza treatment trials patients had to present an ‘influenza-like illness’ defined by the presence of (objective) fever and/or (subjective) feverishness and at least two of the four following symptoms: headache, myalgia, cough and/or sore throat. The primary endpoint, that is, the primary outcome measure in trials, was time to alleviation of influenza symptoms. Alleviation was defined as no fever (temperature < 37.8 °C and/or feverishness as none) and headache, myalgia, sore throat and cough recorded as none or mild, and had to be maintained over the next 24 hours. A number of pre-specified secondary endpoints were also examined, including, but not limited to, complications of influenza and antibiotics use. To mimic the expected real-world use of the drug, patients were allowed to use relief medication (paracetamol and cough suppressant) in an unrestricted fashion.

Although patients were recruited on the basis of symptoms of influenza, patients’ infection status was subsequently determined using diagnostic tests. Besides influenza viruses, a host of other infectious agents can produce influenza-like symptoms. Consequently, trials recruited both influenza positive (IP) and negative patients, and together the IP and influenza negative subpopulations made up the so-called intention-to-treat (ITT) population. In the phase III trials, the percentage of IP patients ranged from 71% to 77% (Table 1). This percentage is expected to be much lower in clinical praxis, possibly around 15%, although the exact percentage will vary between seasons and treatment settings [4]. Crucially, in the absence of an appropriate ‘point of care’ diagnostic test, doctors similarly base treatment decisions on symptoms alone.

**Table 1:** Results on the primary endpoint: median time to symptom improvement (days)

Trial	Southern Hemisphere (NAIB3001)	Europe (NAIB3002)	North America (NAIA3002)
Intent-to-treat	n = 455	n = 356	n = 777
Placebo	6.5	7.5	6.0
Relenza	5.0	5.0	5.5
Difference	1.5	2.5	0.5
95% confidence interval	(0.5 to 2.25)	(0.75 to 3.5)	(–0.5 to 1.0)
*P* value	0.011*	< 0.001***	0.228
Influenza positive	n = 321 (71%)	n = 277 (77%)	n = 569 (73%)
Placebo	6.0	7.5	6.0
Relenza	4.5	5.0	5.0
Difference	1.5	2.5	1.0
95% confidence interval	(0.5 to 2.5)	(1.0 to 4.0)	(0 to 1.5)
*P* value	0.004**	< 0.001***	0.078
High-risk, influenza-positive	n = 52	n = 30	n = 79
Placebo	8.3	11.5	6.0
Relenza	5.0	9.25	6.25
Difference	3.3	2.25	–0.25
95% confidence interval	Not found	Not found	Not found
*P* value	0.16	0.21	0.89

*P < 0.05; ** *P* < 0.01; *** *P* < 0.001

In addition, a ‘high-risk’ influenza positive (HR-IP) population was delineated in trials, defined by age (≥65 years) and/or by underlying chronic condition considered to predispose the patient to a greater risk of a more prolonged and/or severe course of illness.

Table 1 summarises GW’s main analyses in each of the pre-specified populations with respect to the primary endpoint of time to symptom alleviation. The Southern Hemisphere and European studies both showed conclusive effects in ITT and IP populations, and favourable but non-significant point estimates in the HR-IP group. However, despite the larger numbers (n = 777) enrolled in the North America study, results were negative in each of the pre-specified populations. Indeed, for the HR-IP group, the results appear to favour placebo (–0.25 days).

Another critical issue for regulators to address was the drug’s impact on complications of influenza (e.g. pneumonia, bronchitis, sinusitis) and associated antibiotics use. Because GW argued that results in the IP population, but not ITT population, was the best measure of the ‘true efficacy’ of Relenza ([19], p. 30), the company summarised results in the IP population only. Differences were small and favoured Relenza in the IP population in all three trials (Table 2). However, the difference between drug and placebo in reducing complications was only statistically significant in the North American trial, and none of the trials demonstrated a statistically significant reduction in antibiotics use.

**Table 2:** Complications noted and use of antibiotics in influenza-infected patients

Trial	Southern Hemisphere (NAIB3001)	Europe (NAIB3002)	North America (NAIA3002)
Complications			
Placebo	30%	33%	22%
Zanamivir	24%	24%	15%
*P* value	0.24	0.125	0.049*
Antibiotics use			
Placebo	28%	17%	15%
Zanamivir	26%	11%	11%
*P* value	0.58	0.21	0.16

*P < 0.05

### Differences in regulators’ interpretation of key results

Table 3 summarises Swedish/European and United States regulators’ interpretations since 1999 of the effect of Relenza on the duration of influenza symptoms in ITT, IP and HR-IP populations, and Relenza’s capacity to reduce incidences of complications in the IP population, as this was conveyed in product labelling. A number of points can be noted with respect to this comparison of successive United States and European Union product labels for Relenza, including the fact that regulators approved directly contradictory claims with respect to the drug’s efficacy.

**Table 3:** Relenza labelling in Sweden (SWE), European Union (EU) and United States (US), 1999–2017

Label	SWE March 1999	EU June 1999	EU 2001	EU 2017	US 1999	US 2017
Efficacy in IP^a^	Relenza shortens the time of illness with 1–2.5 days compared to placebo	Relenza alleviates the symptoms of influenza and reduces their median duration by 1.5 days (range 1.0–2.5 days)	Label updated to include pooled analysis showing 1.5 day (95% CI 1.0–2.0 days) of shortening of median time of symptoms	Similar statement	Trials in North America suggested up to 1 day of shortening of median time of symptoms compared with placebo, although statistical significance was not reached; in a study conducted in the Southern Hemisphere, a 1.5-day difference in median time to symptom improvement was observed; additional evidence of efficacy was provided by the European study	Similar statement
Efficacy in ITT^b^	No mention	No mention	No mention	The difference in time to alleviation of symptoms was 1.0 day (95% CI 0.5–1.5) in the combined analysis of studies	No mention	No mention
Efficacy in HR groups^c^	In some studies a more pronounced therapeutic effect has been seen in patients belonging to the high-risk groups of elderly patients (≤ 65 years) and patients with some chronic diseases of the heart and lungs, although only a limited number patient in these risk groups have been evaluated	A limited number of HR patients, i.e. elderly and patients with asthma, were included; data are therefore limited in these groups of patients	A limited number of elderly patients were included; data is therefore limited in this group Label updated with conclusive study (1.5 day difference) in IP patients with underlying respiratory diseases	Similar statement	No consistent treatment effect was demonstrated in patients with underlying chronic medical conditions, including respiratory or cardiovascular disease	Similar statement
Complications^d^	No mention	No mention	Label updated with pooled analysis in the IP population showing significant reduction in incidence of complications and antibiotics use	Similar statement	No consistent differences in rate of development of complications were observed	Similar statement

^a^Efficacy in IP population: United States labels have consistently been the most cautious. Furthermore, unlike European labels, the United States labels do not combine the results from trials, neither as an interval of expected effects, as in the original Swedish label (i.e. 1–2.5 days), nor by pooling studies, as in subsequent European Union labels

^b^Efficacy in ITT population: Although the intended users are patients with influenza-like symptom (i.e. ITT subjects), labels consistently cite results in the IP group. The current Europe-wide label is the exception, reporting results from a pooled analysis in the ITT population; however, the emphasis in current European Union labelling is still on efficacy in IP subjects

^c^Efficacy in HR-IP groups: The original Swedish label from March 1999 professed that Relenza could be more efficacious in the elderly and some other HR subgroups. This statement was promptly removed and it was not present in the first Europe-wide label from June 1999. However, in 2001, a statement was introduced in the European Union label that efficacy had been demonstrated in patients with mild to moderate chronic airways disease. In contrast, FDA has maintained that no effect has been demonstrated in HR-IP patients, including those with chronic airway disease

^d^Complications: FDA has maintained that no consistent effect on the incidence of complications has been demonstrated. The original Swedish and European Union labels did not make any claims regarding effects on complications. However, since 2001, the Europe-wide label reports on a pooled analysis showing fewer complications with Relenza. Notably, Jefferson et al. [8] have pointed out the exact same contradictory claims in the labels for Tamiflu

*CI* confidence interval, *HR* high risk, *IP* influenza positive, *ITT* intention-to-treat

The following sections address three key questions critical to an evaluation of Relenza’s therapeutic effects, namely drug efficacy in the ITT and IP populations, efficacy in HR-IP groups and the drug’s impact on complications of influenza, and describe how regulators came to their sometimes diametrically opposed interpretations. We show that FDA reviewers, unlike their European (i.e. Swedish) counterpart, (1) rejected the manufacturer’s insistence on pooling efficacy data, (2) remained wary of subgroup analyses and (3) insisted on stringent statistical analyses. These differences also meant that the FDA was less likely to depart from prevailing regulatory and scientific standards in interpreting results.

### Efficacy in the ITT and IP populations

#### The FDA’s position

In accordance with standard FDA practice, a multi-disciplinary team, including statistical and medical reviewers and team leaders, carried out the clinical appraisal. This involved independent, statistical analysis of key results as well as detailed qualitative evaluations of trial documentation, consistent with a penetrating and meticulous regulatory review methodology*.* Overall, the DAVP review team judged both Southern Hemisphere and European studies to show relevant therapeutic effects. However, because the North American study had only small and statistically inconclusive point estimates, despite being very well powered, this trial was considered to be negative (Table 1) [20].

Further, DAVP reviewers considered it especially problematic that the pivotal North American study was unsuccessful, because this meant that it was “*a dilemma to try to determine a treatment effect generalisable to a North American population*” ([21], p. 2), i.e. the population most germane to the FDA’s jurisdiction.

Adding fuel to the fire, Dr Michael Elashoff, the DAVP’s statistical reviewer, also argued that, since the proportion of IP patients was very high (more than 70%) compared to the population that would be prescribed the drug (likely less than 20%), the trials markedly overstated Relenza’s real-world benefit [22]. However, this critical remark did not resonate with Elashoff’s colleagues on the clinical side. Rather, the DAVP medical reviewer, Dr Barbara Styrt, concurred with GW that effects in the IP population were the most relevant because this addressed the drug’s activity on the target disease [20]; hence, DAVP’s efficacy appraisal centred almost exclusively on results in IP patients, consistent with the view codified in subsequent FDA guidance documents on anti-influenza drugs [23].

When DAVP asked GW to explain the negative results in the North American trial, the company responded that the North American study should be viewed not as negative but as less positive. GW’s main argument was that effects approached statistical significance (*P* = 0.078; Table 1). However, this reasoning did not convince the DAVP statistics team leader who noted that, while *P* = 0.078 might be interpreted as a “*trend in the right direction*” ([24], p. 1), independent analyses by Dr Elashoff using alternative statistical tests or endpoints showed this interpretation to be inappropriate as this trend was not replicated in most analyses, and therefore could not be regarded as robust [22]. The DAVP’s position followed, therefore, from their independent statistical analysis that went beyond the manufacturer’s analyses.

Additionally, GW collated a summary list of “*statistically significant results in favour of zanamivir*” from the North American trial that, the company argued, were supportive of a therapeutic effect in this trial ([20], p. 18–9). This list comprised evaluations on a number of secondary endpoints (some retrospectively defined), including cough, time with fever, average maximum daily temperature, investigator’s assessment of symptoms post-treatment and complications of influenza. However, Dr Styrt, who reviewed this new submission, was unconvinced about the statistical and clinical relevance of these secondary analyses. In her view, any statistically significant results in favour of Relenza from the North American trial needed to be assessed in the context of the multiple hypothesis testing performed and the risk of spurious findings, and in relation to results obtained in the other, clearly positive studies. Dr Styrt therefore concluded: “*the usefulness of this P value for ascribing importance to a small proportion of the large number of secondary analyses appeared limited*” ([20], p. 19).

An alternative way of analysing the data – which, as we discuss in detail below, was endorsed by the Swedish regulator – would have been to pool efficacy data from the three pivotal trials. Notably, pooling would have ‘cancelled out’ the negative North American study while simultaneously producing a statistically significant overall effect of 1.5 days in the pooled IP population (and 1.0 day in the ITT population).

However, at an early point in the appraisal process, the DAVP turned down offers by GW to pool efficacy data on the grounds that independent assessment by DAVP reviewers showed there to be “*too much statistical and clinical heterogeneity to justify this approach*” ([20], p. 144) – consistent with the establishment of company-independent opinions among reviewers regarding the appropriate way to analyse and interpret trials. For example, according to Dr Elashoff, there was empiric evidence that the efficacy in the foreign studies could not be extrapolated to the United States population as he could show a statistically significant treatment-by-study interaction, suggesting that the disparate trial results were unlikely to be random fluctuations but represented real differences [22]. Furthermore, Dr Elashoff noted how there was a sensible explanation for the disparate results, namely that use of relief medication was almost twice as high in the negative North American study compared to the clearly positive European study, with the Southern Hemisphere study laying in between [22].

Similarly, Dr Styrt’s clinical review drew attention to the fact that the European study “*appeared uniquely isolated at the particularly high effect estimate*”, and therefore argued it would be inappropriate to combine results with other studies ([20], p. 144). Moreover, her report said the Southern Hemisphere study differed from the North American and European studies in too many respects, “*most notably the lack of an objective temperature criterion for entry, the requirement that symptoms be present for no more than 36 hours at entry, use of different direct tests for influenza diagnosis…and a shorter duration of symptom recording*” ([20], p. 13) (in the Southern Hemisphere study symptom recoding was not continued beyond the first 2 weeks).

The DAVP reviewers concluded that, taken together, this level of statistical and clinical heterogeneity meant pooling would likely result in misleading estimates of treatment effects and their level of confidence, especially in relation to the United States population. Efficacy appraisal was, therefore, to be based on analysing each study separately. Notably, in advancing this argument, DAVP reviewers aligned themselves with existing agency thinking regarding the pooling of efficacy data [25], which, in turn, were based on influential tripartite (United States, European Union and Japan) International Congress on Harmonisation regulatory guidelines regarding statistical analyses of clinical trials [26]. Specifically, whilst acknowledging the potential value of combining results from similar trials for improving precision in estimation of effects, FDA guidance emphasised that pooling should be pre-specified (as opposed to retrospective, as in the present case), and that “*attention should be paid…to the homogeneity of their* [the trials’] *results, and to the proper modelling of the various sources of variation*” ([25], p. 39).

Thus, why did the FDA approve Relenza despite reviewers’ negative opinions on the product’s proven efficacy in the United States population? An opinion mirrored by a distinctly negative recommendation by the DAVP’s own Advisory Committee [27], whose members voted 13-4 against recommending approval. Space constraints do not allow us to go into the details (but see authors, forthcoming), but one important argument was “*the public health need*” for a new anti-influenza drug in light of the risk of viral resistance developing against available products (rimantidine and amantadine) [28]. However, because of lingering concerns about Relenza’s efficacy in the United States population, the FDA inserted two important qualifications in the label, namely (1) that results in the North American study were not statistically significant, and (2) that the magnitude of treatment effect varied between studies, with possible relationships to the amount of relief medication used (Table 3).

#### The MPA’s position

Dr Ingrid Uhnoo, an MPA infectious disease expert, evaluated the clinical part of the Relenza submission. Like the DAVP medical reviewer, Dr Uhnoo considered the IP population most relevant for efficacy evaluation since this tested the drug’s effect on the biological disease [29]. Furthermore, Dr Uhnoo’s overall valuation of trial results was akin to DAVP’s assessment. She had few concerns regarding the Southern Hemisphere and European studies results, but viewed the North American study as essentially negative. However, unlike the DAVP statisticians, she did accept that there were some “*trends in favour of zanamivir*” in the North American trial ([30], p. 15).

Notably, and in sharp contrast to FDA appraisals, the MPA did not (and still do not) generally conduct statistical re-analyses of data sets (written communication to author: Ingrid Landberg, Director of Efficacy and Safety Unit at MPA, November 2016). Nor did the MPA normally involve a statistician in the primary review, although a statistician sat on the agency’s Board that reviewed all MPA expert assessments (author interview with Senior MPA Statistician). This followed MPA standard operating procedure, consistent with the institutionalisation of a less probing review methodology compared to the FDA. A consequence of this was that the MPA, in evaluating Relenza, did not, for example, check the statistical robustness of results, conduct exploratory analyses or test for treatment-by-study interaction, as DAVP had done. In addition, MPA’s clinical review was also significantly less exhaustive than DAVP’s evaluation (see below).

Notwithstanding its more superficial review, the MPA’s clinical assessment resulted in 42 questions that GW was asked to respond to [30]. With respect to efficacy, two questions pertained to the negative North American study results, which, the MPA stated, “*need to be analysed in depth*” ([30], p. 33).

GW’s response mirrored their response to the DAVP; the company did not view the North American study as negative, “*but as less strongly positive than the other pivotal studies*” ([31], p. 31). To support this contention, GW submitted, as they did to the DAVP, a list of ‘secondary measures of efficacy’ from the North American study displaying statistically significant treatment benefits favouring Relenza. With respect to explaining outcome differences between the North American study and the other studies, GW commented that, “*Any consistent differences in observed efficacy are more likely to be due to cultural differences in symptom reporting and relief medication use*” ([30], p. 33).

However, unlike the DAVP medical reviewer who had thoroughly scrutinised and criticised GW’s explanations, the MPA assessor appears to have been uninterested or unable to pursue the issue further, and wrote: “*The applicant has satisfactory* [sic] *analysed the results of study NAIA3002* [North American study] *and the potential determining factors for the decreased treatment response observed*” ([32], p. 2). The ‘point’ was thus considered “*resolved, although no new data to explain the difference in efficacy outcome of study NAIA3002 were submitted*” ([30], p. 33).

Once the issue of the inconclusive North American study was judged to be ‘resolved’, the MPA went on to formally conclude that, overall, the evidence showed that Relenza alleviated symptoms of influenza 1–2.5 days quicker than placebo, which justified marketing authorisation [30]. This implied that the MPA – in agreement with GW [19] – saw results from the three trials as representing a range of expected effects (i.e. 1, 1.5 and 2.5 days, respectively). Importantly, this interpretation diverged from the DAVP’s position that there was too much statistical and clinical heterogeneity to permit such a generalisation.

The evidence suggests, therefore, that the issue of inter-trial heterogeneity, for example, related to differential frequency of relief medication use, did not overtly trouble the Swedish regulator, at least not so as to preclude combining efficacy data from different studies. As further evidence of this – and despite Dr Uhnoo conceding that “*there were some differences between trials in inclusion criteria with respect to age, duration of influenza symptoms at entry and also to the definitions of absence of fever*” ([30], p. 8) – she concluded that pooling was justified because of the similarities between studies ([33], p. 33). Subsequently, in 2001, and on the basis of the MPA assessor’s recommendations, European regulators allowed GW to include in the Europe-wide product label the results from a pooled efficacy analysis, showing a statistically conclusive effect of 1.5 days in the IP population, alongside results from the individual studies (Table 3) [33].

It is important to note that this pooling of efficacy data was not only incongruent with the FDA’s position, but was also incongruent with the official European Medicines Agency’s (EMA) bio-statistical guidelines from 2001 [31]. According to these guidelines – which, like the FDA guidance, drew on International Conference on Harmonisation guidelines [26] – retrospective pooling “*should be avoided*” ([31], p. 2). Furthermore, EMA guidelines specified that pooling should not be used to reconcile conflicting results from positive and negative studies. In ‘exceptional situations’ retrospective pooling could be allowed (because “*meta-analysis seems to be the only way to provide reliable proof of efficacy*”), but regulatory prerequisites included, among other things, “*no statistically significant heterogeneity*” between trials and “*inconclusive studies showing positive trends in the primary variable*” ([31], p. 3), which was arguably not the case for Relenza trials.

At the same time, it should be emphasised that the MPA’s decision to approve Relenza was not based on this pooled analysis. Rather, pooling had implications for how results were conveyed in the labelling. Nevertheless, together with the evidence presented below, the tolerance towards retrospective pooling and the apparent disregard for inter-trial heterogeneity suggests a tendency on the part of the MPA to depart from prevailing regulatory standards when interpreting the drug’s beneficial effects.

### Efficacy in high-risk groups

#### The FDA’s position

The DAVP were unimpressed by the drug’s efficacy in HR-IP patients (e.g. the elderly and patients with certain chronic disorders) and reviewers highlighted numerous problematic issues related to the studies’ design, conduct and results. As with efficacy in the IP group as a whole, statistical heterogeneity between trials was considered a problem for interpreting results in the HR-IP group [20]. A particular concern was that, whilst point estimates of time to symptom alleviation were shorter with Relenza (albeit not statistically significant) in the European and Southern Hemisphere trials, in the North American study, which had recruited the largest number of HR-IP patients, this group did numerically worse with Relenza (–0.25 days) (Table 1).

DAVP also highlighted how the definition of ‘high-risk’ differed between studies, potentially creating important clinical heterogeneity between studies. Specifically, only in the Southern Hemisphere study were HR categories defined to include metabolic/endocrine disorders and immune compromised patients in addition to those with chronic respiratory and cardiovascular disease (Table 4) ([20], p. 31). Moreover, reviews of individual patient case report forms by DAVP staff suggested that, even within studies, “*high-risk definitions might in some instances be applied differently by different investigators, so that uniformity in the average severity of underlying disease of these subgroups in different studies could not be assumed with confidence*” ([20], p. 49). In sum, Dr Styrt’s medical review concluded that “*results for the ‘high risk’ subgroup may require some caution in interpretation*” ([20], p. 39).

**Table 4:** Median days to alleviation in high-risk subgroups

Trial	Southern Hemisphere (NAIB3001)	Europe (NAIB3002)	North America (NAIA3002)
Elderly	n = 9	n = 12	n = 37
Placebo	13	> 26.5	7.25
Relenza	2.75	11	4.5
Difference^a^	10.25	> 15.5	2.75
Cardiovascular	n = 3	n = 6	n = 19
Placebo	8.5	16.5	7
Relenza	1.5	21	7.5
Difference^a^	7.0	–4.5	–0.5
Respiratory	n = 41	n = 17	n = 36
Placebo	7	10.75	5.5
Relenza	5.5	6.5	8.75
Difference^a^	1.5	4.25	–3.25
Endocrine/metabolic	n = 6	N/A	N/A
Placebo	13		
Relenza	4		
Difference^a^	9		
Immune compromised	n=2	N/A	N/A
Placebo	4.5		
Relenza	3		
Difference^a^	1.5		

^a^ Differences are statistically non-significant

Besides concerns about differences in enrolment criteria and definitions across and within trials, DAVP staff worried that the HR group included subjects with quite distinct risk factors that “*cannot be assumed to have a uniform effect on the course of influenza or its response to treatment*” ([20], p. 49). One way of addressing this issue would have been to analyse results in HR-IP subgroups (e.g. chronic respiratory disease) by retrospectively pooling data from several studies to increase statistical power. However, according to Dr Styrt, this was inappropriate as detailed examination of results and trial protocols showed “*there was not enough consistency across studies to suggest that pooled analyses could be used with confidence*” ([20], p. 54).

Relatedly, DAVP rejected GW’s suggestion to pool the results from all HR-IP individuals. GW presented a treatment-by-study interaction analysis with a *P* value of 0.128 as a demonstration that “*there is no specific contraindication to pooled…analysis, across protocols*” ([20], p. 119). However, this argument was rejected by DAVP on the grounds that “*the absence of a statistical interaction is not generally considered to prove homogeneity across studies, especially in the context of the many differences between studies…*” ([20], p. 119).

In sum, DAVP concluded that there was no compelling evidence of efficacy in the HR-IP population due to the small, inconclusive differences (generally favouring Relenza), and the substantial statistical and clinical heterogeneity. It is important to note how this view followed from DAVP’s meticulous and detailed engagement with the quantitative and qualitative facets of trials.

#### The MPA’s position

Like the DAVP, the MPA assessor, Dr Uhnoo, lamented the small number of HR-IP patients recruited, noted the statistically inconclusive results in this group across trials, and expressed concerns that, in the North American study, which had recruited the largest number of high-risk subjects, Relenza patients fared worse than patients on placebo [30]. She did not, however, further explore the underlying clinical heterogeneity (for example, by examining patient case report forms to ensure consistency in the definition of ‘high-risk’ within and across trials) as was done by the DAVP [30].

Moreover – and again unlike the DAVP team – she seemed inclined to view the evidence as supporting efficacy in HR-IP patients, noting that the evidence “*is considered very limited, but do indicate treatment benefit*” ([32], p. 3). Dr Uhnoo also referred to “*ongoing studies in the high risk patients*”, which would “*probably clarify this issue*” ([30], p. 35). However, rather than wait for completion of those studies to resolve remaining uncertainties, the MPA concluded that “*Results suggest a slightly more pronounced effect* [in the high risk group] *on the duration of illness with an average shortening of 2.5 days (variation 2.25-3.25 days)*” ([34], p. 7). It is important to note that these point estimates referred to the results in the Southern Hemisphere and European studies (*P* = 0.16 and *P* = 0.21, respectively), but disregarded the negative point estimate in the North American study. Thus, the MPA seemed willing to give the drug (and the manufacturer) significant benefit of the doubt with respect to proving efficacy in the HR-IP group, despite recognising the paucity of data and the statistically inconclusive results – some of which actually favoured placebo.

The MPA took a similarly permissive approach when it accepted GW’s retrospective analyses of HR-IP patient subgroups (e.g. chronic respiratory disease), despite this involving multiple analyses, each with a small number of patients, where apparent efficacy differences might easily arise due to chance. Thus, the original Swedish Relenza label from March 1999, written by GW and approved by the MPA, contained the following statement:

“*In some studies, a more pronounced treatment effect has been seen in patients that belong to the risk group of elderly patients (≤65 years) and patients with certain chronic disorders in heart and lungs, although only a limited number of patients in these risk groups have been evaluated*.”

The claim that Relenza exhibited “*a more pronounced treatment effect*” on the elderly appears to refer to the positive, but statistically inconclusive, point estimate in elderly subjects in each of the pivotal trials (Table 4). By contrast, it is difficult to understand how the statement that Relenza showed “*a more pronounced treatment effect in patients with heart and lung disorders*” was derived since point estimates for the high risk cardiovascular group in two out of three phase III studies favoured placebo. In fact, this claim was subsequently removed from the drug’s label, and was not present in the European harmonised label (Table 3), which suggests it was removed during negotiations with other European regulators on how the drug’s properties should be conveyed.

Nevertheless, in 2001, the European label was updated with a statement that efficacy had been demonstrated for certain high risk groups – a claim that has remained in European labels (Table 3). The basis for this new claim was a novel trial, NAI3008, which showed a statistically significant reduction of 1.5 days on the duration of influenza symptoms in IP patients with mild to moderate chronic respiratory disease [33]. Interestingly, there is no evidence from publicly available documents that GW similarly submitted results from NAI3008 to the FDA with the purpose of updating the United States label. Indeed, to this day, FDA maintains that no treatment effect has been demonstrated in patients with underlying chronic medical conditions, including respiratory disease (Table 3).

GW also hoped to include a new claim in the European Union product label that Relenza benefited patients across all HR-IP subgroups, and therefore submitted a pooled efficacy analysis of HR-IP patients from the now four principal trials (i.e. NAI30008 plus the original three) demonstrating an overall reduction in duration of influenza symptoms in HR-IP patients (1.5 days difference, *P* = 0.005) [33]. As the ‘reference country’ in the European Union, the MPA continued to serve as the evaluating agency with Dr Unhoo as the assessor. According to her understanding, the pooled analyses provided further support of efficacy in HR-IP patients. However, she also noted that significant efficacy was not demonstrated in the small subgroup of elderly patients, where a pooled analysis failed to reach statistical significance [33]. This contradicted earlier statements approved in labelling (see above). GW’s position on this issue, however, was that “*it is not practicable to perform clinical studies to demonstrate efficacy (with statistical significance) in all conceivable patient sub-groups*” ([33], p. 36). Rather, they argued, “*a consistency of effect has been demonstrated, from which it is reasonable to assume efficacy across sub-groups*” ([33], p. 36). Nevertheless, this argument did not persuade the MPA assessor, who noted that efficacy analysis from an on-going study (NAI30012) in the elderly population “*is awaited before a conclusive statement could be made*” ([33], p. 36). It is interesting to note how this attitude of requiring statistically conclusive results, and of not accepting claims to efficacy prior to the completion of on-going studies, differed from the MPA’s earlier permissive regulatory attitude, perhaps suggesting learning from past experiences.

Unfortunately for GW, and further contradicting of MPA’s initial statement that Relenza would work better on elderly patients, study NAI30012 failed to demonstrate a beneficial treatment effect. Consequently, since 2001, labelling in the European Union has cited the positive effects asthma/chronic obstructive pulmonary disease patients derived from study NAI30008, but simultaneously noted that efficacy has not been established in elderly patients.

### Complications of influenza

#### The FDA’s position

To this day, FDA maintains that the evidence submitted to them does not prove a beneficial effect of Relenza on the incidence of complications. In reaching this judgment, the DAVP team pointed to the same kind of technical and methodological issues that had troubled reviewers with regard to effects in the HR-IP group, i.e. the small, inconclusive results (generally favouring Relenza) alongside important heterogeneity, which contraindicated pooling [20].

In addition, DAVP argued the trials were not individually powered to demonstrate differences, as the percentage of subjects categorised as experiencing complications was relatively low (Table 2). The one study in which complications in the IP population were ‘significantly’ less frequent with Relenza was the otherwise negative North American study (*P* = 0.049; Table 2). However, as we have seen, DAVP did not trust any ‘significant’ finding from the North America trial because of the problems of multiplicity and the lack of consistency in analyses [20].

Furthermore, Dr Styrt noted that there appeared to be differences in how complications were recorded across studies [20]. For example, detailed DAVP analyses of patient case report forms showed that check-boxes used for recording events were not uniform across trials. Another concern was the “*disparate components of the ‘complication’ definition*” ([20], p. 49); in particular, that “*some complications of influenza (such as pneumonia or respiratory failure) would usually be regarded as more serious than others (such as sinusitis or pharyngitis)*” ([20], p. 46). Therefore, Dr Styrt argued, it was reasonable to examine occurrence of individual types of complications in addition to the aggregate analysis, but, unfortunately, “*numbers were small for any individual complication type and no firm conclusion could be derived with regard to either increases or decreases in specific complications associated with treatment assignment*” ([20], p. 48).

#### The MPA’s position

Like DAVP reviewers, the MPA assessor acknowledged that studies were not individually powered to demonstrate conclusive results on complications. Nonetheless, as with effects in HR-IP patients, she seemed inclined to view the totality of results as still providing adequate evidence of effect, albeit without exploring to any great extent the heterogeneity in trials, for example, by looking at instructions for how complications were recorded [30, 33].

While the alleged evidence of a beneficial effect was not considered sufficiently strong or important to merit mentioning in the original Swedish label, the MPA’s lengthier product monograph (a post-authorisation summary text aimed at health professionals) included the following description of effects on complications:

“*The most common complications were bronchitis, sinusitis and otitis media, which in one study were significantly less frequent with zanamivir treatment. Overall in the Phase III trials, the complication rate was lower and there was less use of antibiotics in patients treated with zanamivir compared with placebo*” [35]*.*

Notably, the one study in which “*complications…were significantly less frequent*” [35] with Relenza was the otherwise negative North America study, hence adding to the evidence of a less stringent attitude towards statistics from the Swedish regulator compared to the DAVP.

Regarding the second claim of an overall reduction in complication rate and antibiotics use, this judgment was based on a pooled analysis conducted by GW ([36], p. 15–6). Indeed, in 2000, the MPA agreed to recommend updating the European label with the results of this pooled analysis showing statistically significant reductions in complications (29% vs. 22%; *P* = 0.004) and use of antibiotics (19% vs. 14%, *P* = 0.021), compared to placebo ([33], p. 34). Thus, as with the labelling decisions regarding Relenza’s effect on time with influenza symptoms, divergent regulatory interpretations of Relenza’s effects on complications boiled down to judgments on whether pooling of efficacy data was appropriate or not.

### Explaining regulatory divergence

The immediate explanation for these divergent regulatory interpretations of Relenza’s efficacy was that DAVP, but not MPA, rejected the pooling of efficacy data, remained wary of subgroup analyses and insisted on stringent statistical analyses. Notably, in comparison to the DAVP, the MPA repeatedly took a more permissive approach, and was more likely to depart from prevailing regulatory and scientific standards with respect to the evaluation of the design, conduct, analysis and interpretation of the data. For example, the MPA displayed a greater willingness to depart from established scientific interpretive norms by accepting subgroup analyses within the HR-IP group and a ‘significant’ effect on complications in the North American study despite the well-known risks of spurious associations with multiple hypothesis testing [37]. The MPA, and by extension other European regulators accepting the MPA’s assessment, also allowed themselves to deviate from joint European Union regulatory standards [31], for example, with respect to GW’s retrospective pooling of efficacy data.

However, why did regulators’ methods and criteria differ in this way? Our analysis suggests links between regulatory outcomes (in the form of divergent product labelling) on the one hand and, on the other, regulatory resources, disciplinary expertise and institutionalised review methodologies and practices. Thus, DAVP benefited from having greater resources, which included a review team made-up of statistical and clinical reviewers who had sufficient time and the multi-disciplinary expertise required to conduct critical, in-depth assessments. This allowed its reviewers to collectively develop ‘deep’ knowledge [38] concerning the clinical and statistical facets of trials, and informed opinions regarding suitable methods for analysing trial results.

By contrast, the single, clinical assessor for the MPA did not perform independent statistical analyses of raw data or cross check various definitions and their application within and between trials. Instead, she relied largely on summary tables of data and reports compiled by the drug’s sponsor, resulting in a more superficial understanding of the evidence pertaining, for example, to differences in how study definitions of ‘risk’ or ‘complications’ were applied. Arguably, this superficial understanding weakened the MPA assessor’s ability to challenge the manufacturer’s proposed methods for analysing results (i.e. pooling, subgroup analyses) or problematic interpretations of the data (e.g. that the North American trial showed a conclusive effect on complications). It may also have encouraged a more permissive regulatory approach – a permissiveness that is perhaps most evident in the MPA’s acceptance of GW’s claim that patients with cardiovascular disease benefited from Relenza, when results in two out of three studies actually favoured placebo.

Alongside these important differences in regulators’ methodologies and conclusions, our analysis also points to similarities. The elephant in the room was whether judgments were to be derived from results in the ITT or IP populations and, related to this, how to deal with the abnormally high number of IP patients in trials (over 70%). Regulators on both sides of the Atlantic concurred with the company that the IP population was most relevant for efficacy evaluation, since this tested the drug’s effects on the disease. By contrast, some researchers [38] and Health Technology Assessment agencies [4] have argued that, from a public health perspective, results in the ITT population provide a better, albeit still inflated, approximation of efficacy, since this population is more similar to the real-world patient population.

Yet, we argue that, even from the regulators’ perspective, to rely on results from the IP population was questionable since the drug was intended for patients with influenza-like symptoms, rather than only those infected by influenza virus. Furthermore, since the label constitutes the factual basis for drug marketing, emphasising effects in the IP population in the label meant regulators authorised the use of these estimates in marketing. How this might be a problem became evident after the MPA’s unit for drug information, tasked with overseeing drug marketing, complained to the Swedish pharmaceutical industry’s self-regulatory body governing drug promotion [39] about a promotional claim that Relenza shortens the time of illness by 1–2.5 days compared to placebo. MPA’s objection was that the claim could mislead doctors since efficacy is likely inferior in clinical practice where the proportion of IP patients is typically low [40]. Tellingly, GW successfully challenged the MPA allegation on the grounds that their marketing material simply conveyed information approved by MPA in labelling.

## Discussion

### The importance of ‘deep’ product reviews

Our observation that the MPA relied largely on summaries written by the sponsor whilst the DAVP conducted a more thorough assessment, including independent examination of trial documentation and statistical analysis of raw data, is not unique to this case but has been noted in previous social scientific studies of the FDA and national and supranational drug regulatory agencies in the European Union [41, 42]. One important insight emerging from this body of work is that the FDA and European regulators have developed very different review methodologies – enabled and constrained, in turn, by different levels of funding and agency resources. These diverse review methodologies are rooted in distinct institutional cultures and histories [42–45], and continue to be relevant, judging from a recent report by the Swedish National Audit Office alleging that the MPA – the agency that in 2015 handled the largest number of EMA evaluations – lacks resources and displays an inappropriate trust in manufacturers’ data analyses [46].

It is important to acknowledge that European regulators’ less probing evaluative methodology does not always translate into permissive regulation and, conversely, that FDA’s ‘deep’ review does not always ensure a precautionary approach [41]. Nevertheless, the FDA’s greater attention to the qualitative and statistical facets of trials, and reviewers’ sceptical attitude towards pooling and multiple hypothesis testing, meant that the United States regulators consistently approved more conservative, and arguably more accurate, Relenza labelling statements than European regulators. Thus, our analysis shows that FDA reviewers’ more comprehensive evaluation meant that they were more able, and willing, to challenge problematic company analyses.

This finding has important implications for on-going policy debates – particularly in the United States – relating to the speed of regulatory review. Following decades of criticism by the pharmaceutical industry and industry-funded think-tanks that the FDA was overly slow and bureaucratic [47], the agency now boasts some of the fastest drug review times in the world, as evidenced by comparative studies in the medical and policy literature by researchers attempting to defend the FDA from further attempts to whittle down Congress-mandated regulatory review times [48–50]. However, as one United States patient group commented, and as our analysis demonstrates, regulatory ‘success’ should not be measured solely by the rapidity of an agency’s review process but, rather, “*by the completeness and scientific soundness of its work*” ([47], p. 62). Thus, one of the lessons of the Relenza case is that adequate resources and an institutional culture conducive to a critical and rigorous regulatory science are as, if not more, important than meeting strict review deadlines. Moreover, analyses that compare different agencies’ overall review times may not reflect the actual time that reviewers are engaged in scrutinising company submissions. Policy debates and academic research comparing FDA and EMA timelines may inadvertently put pressure on the EMA to further reduce the time assessors spend reviewing marketing authorisation applications, with potentially negative impacts on public health.

### Upgrading the public health role of regulatory science

We have shown that the FDA’s probing and meticulous review practices in the case of Relenza resulted in ‘deep’ knowledge of the clinical and statistical aspects of the existing evidence base for the drug and, arguably, a more accurate picture of the nature and magnitude of its health benefits. Such knowledge is a valuable asset, but this asset can only be realised if the information is widely communicated to and understood by the public health community [51]. Yet, a striking and constant feature of the conflicting meta-analyses and heated controversies concerning the usefulness, cost-effectiveness and stockpiling of NIs has been the determined lack of engagement by national and supranational drug regulatory agencies in these public debates.

To illustrate this, one can point to the fact that, after researchers ‘discovered’ regulatory assessments differed between countries yet were unable to explain the reason for these divergences [2], neither the FDA, the MPA, nor the EMA made their positions sufficiently clear. Indeed, as debates in the medical literature raged on, a peculiarity of these disagreements was that protagonists on both sides referred to the FDA’s judgment to substantiate conflicting positions. Thus, Jefferson et al. of the Cochrane collaboration [8] stress that their meta-analyses of all pre- and post-marketing trials confirm the FDA’s conservative conclusions, whilst Monto et al. [52] – promoting the use of NIs to reduce the risk of complications – cite the FDA to support their view that efficacy should be evaluated in the IP population. None of these authors refer to the FDA’s refusal to accept pooled efficacy data. Instead, and despite important differences between these various meta-analyses, a common feature is that they combine pre- and post-marketing (i.e. phase I-IV) trials of variable quality, that display even greater heterogeneity [8] than the three, phase III studies that the DAVP found sufficiently heterogeneous to argue against pooling. Open discussion of the agency’s position with respect to pooling was thus a critical, but missing, part of the debate – something the FDA did nothing to correct.

Doshi [51] has recently argued that regulators are in a unique position with respect to their detailed understanding of the quality and quantity of the evidence base for new drugs, and points to a number of cases in which regulatory knowledge existed to challenge inappropriate reporting of trial data in the medical literature. A further example, not referenced by Doshi, is a pooled analysis in the *New England Journal of Medicine* in 1997 of two phase II studies for Relenza (NAIA2005 and NAIB2005), which states that the definition of illness included “*the presence of fever*” in both trials [53]. However, when these trials were submitted to the FDA for review, DAVP reviewers, unlike the MPA assessor [30], dismissed pooling because their investigation of original clinical study reports and protocol material had unearthed important differences between trials, including different inclusion criteria for ‘fever’ in studies and partly different laboratory methods for confirming influenza ([20], p. 64), and because the analysis involved multiple hypothesis testing ([20], p. 70).

This discussion, then, raises important policy questions, not only about the most effective practices and methods of regulatory science, but also about the public health role of regulators and what that role should entail. In 2009, the new Commissioner of the FDA wrote in the *New England Journal of Medicine* that establishing the FDA as a public health agency required the organisation to communicate “*frequently and clearly about risks and benefits*”, “*provide the data on which it bases its regulatory decisions… and explain its decision-making process to the public*” and foster “*a culture that encourages scientific exchange*” [54]. Just a few months later, a paper in the *British Medical Journal* described the maze of contradictory statements about NI’s efficacy made by researchers, industry, regulators and other public bodies [2]. Yet, regulatory bodies failed to engage in a ‘scientific exchange’ in 2009, nor did they enter the fray in 2014 when the controversy erupted again after the Cochrane collaboration published meta-analyses of all Relenza and Tamiflu trials showing that that the drugs’ efficacy had been much overstated, in part because of major reporting and publication biases [8].

We would argue that persistent uncertainties and knowledge deficits regarding NIs could have been ameliorated had regulators explained their contrasting methodologies and judgments (in addition to making all the raw data available from submitted trials [2]). Had regulators, and particularly the FDA, effectively disseminated their gained knowledge about the qualitative and statistical facets of trials alongside their methodological deliberations, this would most likely have improved the synthesis and critical appraisal of the clinical evidence by the broader medical community [38]. That being said, the broader medical community, including authors of research papers and systematic reviews, journal editors and reviewers, and clinicians, must also accept some responsibility for the underutilisation of the regulatory reviews that have been readily available. Regulators use important economic and intellectual resources to evaluate drugs, but if regulators’ appraisals and methodologies are not effectively disseminated and used this means that resources are used inefficiently, as has evidently been the case for NIs.

## Conclusion

The FDA’s more comprehensive evaluative methodology resulted in a ‘deeper’ understanding of the Relenza clinical evidence. Our findings challenge the current emphasis on evaluating regulatory performance mainly in terms of speed of review. Furthermore, had regulators explained their contrasting assessments, this could have improved the broader medical community’s critical appraisal of the evidence base for anti-influenza drugs. Regulators use major resources to evaluate new medicines, but if regulators’ assessments are not effectively disseminated and used, resources are wasted.

## Abbreviations

DAVP: FDA Division of Antiviral Products
EMA: European Medicines Agency
FDA: United States Food and Drug Administration
GW: GlaxoWellcome
HR-IP: high-risk influenza positive
IP: influenza positive
ITT: intention-to-treat
MPA: Swedish Medical Products Agency
NI: neuraminidase inhibitor

## References

[1] Moynihan R (2014). Was the flu drug zanamivir a breakthrough or money for old rope?. BMJ..

[2] Doshi P (2009). Neuraminidase inhibitors—the story behind the Cochrane review. BMJ..

[3] UK House of Commons Committee of Public Accounts. Access to Clinical Trial Information and the Stockpiling of Tamiflu. London: House of Commons Science and Technology Committee; 2014. https://www.publications.parliament.uk/pa/cm201314/cmselect/cmpubacc/295/295.pdf. Accessed 1 June 2017.

[4] Burls A, Clark W, Stewart T (2002). Zanamivir for the treatment of influenza in adults: a systematic review and economic evaluation. Health Technol Assess.

[5] Monto AS, Webster A, Keene O (1999). Randomized, placebo-controlled studies of inhaled zanamivir in the treatment of influenza A and B: pooled efficacy analysis. J Antimicrob Chemother.

[6] Dobson J, Whitley RJ, Pocock S, Monto AS (2015). Oseltamivir treatment for influenza in adults: a meta-analysis of randomised controlled trials. Lancet.

[7] Kaiser L, Wat C, Mills T, Mahoney P, Ward P, Hayden F (2003). Impact of oseltamivir treatment on influenza-related lower respiratory tract complications and hospitalizations. Arch Intern Med.

[8] Jefferson T, Jones MA, Doshi P (2014). Neuraminidase inhibitors for preventing and treating influenza in healthy adults and children. Cochrane Database Syst Rev.

[9] Jefferson T, Jones M, Doshi P, Del Mar C (2009). Neuraminidase inhibitors for preventing and treating influenza in healthy adults: systematic review and meta-analysis. BMJ..

[10] Ioannidis J (2016). The mass production of redundant, misleading, and conflicted aystematic reviews and meta-analyses. M Quarterly.

[11] Dunn AG, Arachi D, Hudgins J, Tsafnat G, Coiera E, Bourgeois FT (2014). Financial conflicts of interest and conclusions about neuraminidase inhibitors for influenza: an analysis of systematic reviews. Ann Intern Med.

[12] Jefferson T, Doshi P (2014). Multisystem failure: the story of anti-influenza drugs. BMJ..

[13] Lenzer J (2015). Why aren’t the US Centers for Disease Control and Food and Drug Administration speaking with one voice on flu?. BMJ..

[14] Carpenter D, Kesselheim AS, Joffe S (2011). Reputation and precedent in the bevacizumab decision. N Engl J Med.

[15] Abraham J (2008). Sociology of pharmaceuticals development and regulation: a realist empirical research programme. Soc Health Illn.

[16] Abraham J, Lewis G (2000). Regulating medicines in Europe: competition, expertise and public health.

[17] Mäkelä MJ, Pauksens K, Rostila T (2000). Clinical efficacy and safety of the orally inhaled neuraminidase inhibitor zanamivir in the treatment of influenza: a randomized, double-blind, placebo-controlled European study. J Infect.

[18] MIST (Management of Influenza in the Southern Hemisphere Trialists) Study Group (1998). Randomised trial of efficacy and safety of inhaled zanamivir in treatment of influenza A and B virus infections. Lancet.

[19] Glaxo Wellcome (1998). Expert Report on the Clinical Documentation of zanamivir Rotadisksk^TM^ 5mg.

[20] US Food and Drug Administration (1999). NDA 21-036, Medical Officer's Review.

[21] US Food and Drug Administration. NDA 21-036. Record of FDA/Industry Meeting. 1999. https://www.accessdata.fda.gov/drugsatfda_docs/nda/99/021036-admin2.pdf. Accessed 1 June 2017

[22] US Food and Drug Administration. NDA 21-036. Statistical Review and Evaluation. 1999. https://www.accessdata.fda.gov/drugsatfda_docs/nda/99/021036-stats.pdf. Accessed 1 June 2017

[23] US Food and Drug Administration (2011). . Guidance for industry. Influenza: Developing Drugs for Treatment and/or Prophylaxis.

[24] US Food and Drug Administration. NDA 21-036. Statistics Team Leader Memorandum. 1999. https://www.accessdata.fda.gov/drugsatfda_docs/nda/99/021036-stats.pdf. Accessed 1 June 2017

[25] US Food and Drug Administration. Guidance for Industry. E9 Statistical Principles for Clinical Trials. 1998. https://www.fda.gov/downloads/drugs/guidancecomplianceregulatoryinformation/guidances/ucm073137.pdf. Accessed 1 June 2017.

[26] International Conference on Harmonisation of Technical Requirements for Registration of Pharmaceuticals for Human Use (ICH). Harmonised Tripartite Guideline. Statistical Principles for Clinical Trials. 1998. http://www.ich.org/fileadmin/Public_Web_Site/ICH_Products/Guidelines/Efficacy/E9/Step4/E9_Guideline.pdf. Accessed 1 June 2017.

[27] US Food and Drug Administration. Summary Minutes. Antiviral Drugs Advisory Committee. 1999. https://www.fda.gov/OHRMS/DOCKETS/ac/99/meeting/3496m1.pdf. Accessed 1 June 2017

[28] US Food and Drug Administration. Division Director Memorandum. NDA: 21-036. 1999. https://www.accessdata.fda.gov/drugsatfda_docs/nda/99/021036-medreview9.pdf. Accessed 1 June 2017.

[29] Medical Products Agency. Relenza Rotadisksk^TM^ 5mg, Inhalation powder. Asp nr 98-0584. Part IVB - Clinical Assessment. Uppsala: MPA; 1999.

[30] Medical Products Agency. Assessment Report to the Applicant’s Responses to the List of Part IV Questions on Zanamivir. Uppsala: MPA;1999.

[31] European Medicines Agency. CPMP/EWP/2330/99: Points to consider on application with 1. Meta-analyses; 2. One Pivotal Study. 2001. http://www.ema.europa.eu/docs/en_GB/document_library/Scientific_guideline/2009/09/WC500003657.pdf. Accessed 1 June 2017.

[32] Medical Products Agency. Addendum Part IVB. Summary of the Assessment Report to the Applicant’s Responses to the List of Part IVB Questions on Zanamivir. Uppsala: MPA; 1999.

[33] Medical Products Agency. Relenza. Powder for Inhalation, Pre-dispensed, 5 mg (zanamivir). Preliminary Variation Assessment Report in the Mutual Recognition Procedure Type II Variation SE/H/180/01/W07. Uppsala: MPA; 2000.

[34] Medical Products Agency. Treatment Recommendation: Treatment of Influenza with Antiviral Products [Behandlingsrekommenadtion: behandling av influenza med antivirala produkter]. 1999. https://lakemedelsverket.se/upload/halso-och-sjukvard/behandlingsrekommendationer/influensa1999.pdf. Accessed 1 June 2017.

[35] Medical Products Agency. Relenza (zanamivir). Infomration from the MPA [Information från Läkemedelsverket]. 1999. https://lakemedelsverket.se/malgrupp/Halso-%2D-sjukvard/Monografier-varderingar/Monografier-Humanlakemedel/Humanlakemedel-Arkiv/Relenza-zanamivir-%2D-nya-indikationer/. Accessed 1 June 2017.

[36] Medical Products Agency (2006). Overview, Overall Conclusion: Relenza, 5 mg/dose, Inhalation Powder, Pre-dispensed. SE/H/180/001/E/01.

[37] Sun X, Ioannidis JA, Agoritsas T, Alba AC, Guyatt G (2014). How to use a subgroup analysis: Users’ guide to the medical literature. JAMA.

[38] Ioannidis JPA, Karassa FB (2010). The need to consider the wider agenda in systematic reviews and meta-analyses: breadth, timing, and depth of the evidence. BMJ..

[39] Zetterqvist AV, Merlo J, Mulinari S (2015). Complaints, complainants, and rulings regarding drug promotion in the United Kingdom and Sweden 2004–2012: a quantitative and qualitative study of pharmaceutical Industry self-regulation. PLoS Med.

[40] The Pharmaceutical Industry’s Information Practices Committee [Nämnden för bedömning av läkemedelsinformation, NBL]. Case 559/00: Medical Products Agency/Glaxo Wellcome. Regarding information on Relenza with contested misleading information about treatment effects [Ärende 559/00: Läkemdelsverket/Glaxo Wellcome. Angående information för Relenza med ifrågasatt vilseledande information om behandlingseffekter]. The Swedish Association of the Pharmaceutical Industry [Läkemedelsindustriföreningen, LIF]: Stockholm; December 29, 2000. http://www.lif.se/etik/ign-och-nbl/detaljer/?id=1778. Accessed 1 June 2017.

[41] Abraham J, Davis C (2009). Drug evaluation and the permissive principle: continuities and contradictions between standards and practices in antidepressant regulation. Soc Stud Sci.

[42] Abraham J, Davis C (2005). A comparative analysis of drug safety withdrawals in the UK and the US (1971–1992): implications for current regulatory thinking and policy. Soc Sci Med.

[43] Ceccoli SJ (2004). Pill Politics: Drugs and the FDA.

[44] Abraham J (1995). Science, Politics, and the Pharmaceutical Industry: Controversy and Bias in Drug Regulation.

[45] Carpenter DP (2010). Reputation and Power: Organizational Image and Pharmaceutical Regulation at the FDA.

[46] Swedish National Audit Office. Safe and effective medicines - how does the State manage pharmaceutical industry influence? (RiR 2016:9). Stockholm: Swedish National Audit Office; 2016. http://www.riksrevisionen.se/en/Start/publications/Reports/EFF/2016/Safe-and-effective-medicines-%2D-%2Dhow-does-the-State-manage-pharmaceutical-industry-influence/. Accessed 1 June 2017.

[47] Davis C, Abraham J (2013). Unhealthy Pharmaceutical Regulation: Innovation, Politics and Promissory Science.

[48] Downing NS, Aminawung JA, Shah ND, Braunstein JB, Krumholz HM, Ross JS (2012). Regulatory review of novel therapeutics - comparison of three regulatory agencies. New Engl J Med.

[49] Downing NS, Zhang AD, Ross JS (2017). Regulatory review of new therapeutic agents - FDA versus EMA, 2011–2015. New Engl J Med.

[50] Roberts SA, Allen JD, Sigal EV (2011). Despite criticism of the FDA review process, new cancer drugs reach patients sooner on the United States than in Europe. Health Aff (Millwood).

[51] Doshi P, Godlee F (2017). The wider role of regulatory scientists. BMJ..

[52] Monto AS, Dobson J, Pocock S, Whitley RJ (2015). Oseltamivir for influenza – Authors’ reply. Lancet.

[53] Hayden FG, Osterhaus A, Treanor JJ (1997). Efficacy and safety of the neuraminidase inhibitor zanamivir in the treatment of influenza virus infections. N Engl J Med.

[54] Hamburg MA, Sharfstein JM (2009). The FDA as a Public Health Agency. N Engl J Med.

